# Efficacy and safety of ciprofol versus propofol for the induction of anesthesia in adult patients: a multicenter phase 2a clinical trial

**DOI:** 10.1007/s11096-022-01529-x

**Published:** 2023-01-21

**Authors:** Qianmei Zhu, Zhen Luo, Xia Wang, Dongxin Wang, Jun Li, Xinchuan Wei, Jun Tang, Shanglong Yao, Wen Ouyang, Wensheng Zhang, Yunxia Zuo, Xiao Wang, Jin Liu

**Affiliations:** 1 Department of Anesthesiology, West China Hospital, Sichuan University, No. 37 Guoxue Lane, Wuhou District, Chengdu, 610041 China; 2 Department of Anesthesiology, Peking University First Hospital, Beijing, 100034 China; 3 Department of Anesthesiology, The 2nd Affiliated Hospital and Yuying Children’s Hospital of Wenzhou Medical University, Wenzhou, 325027 China; 4 Department of Anesthesiology, Sichuan Provincial People’s Hospital, Chengdu, 610072 China; 5 Department of Anesthesiology, Shanghai Fifth People’s Hospital, Fudan University, Shanghai, 200240 China; 6 Department of Anesthesiology, Union Hospital, Tongji Medical College of Huazhong University of Science and Technology, Wuhan, 430022 China; 7 Department of Anesthesiology, The Third Xiangya Hospital, Central South University, Changsha, 410013 China

**Keywords:** Anesthesia, Ciprofol, Propofol, Sedation, Tracheal intubation

## Abstract

**Background:**

Ciprofol is a novel 2, 6-disubstituted phenolic derivative anesthetic that binds to the gamma-aminobutyric acid-A receptor.

**Aim:**

To determine the equally potent dose of ciprofol compared with propofol as an induction agent for general anesthesia in patients undergoing selective surgery, and to assess its safety.

**Method:**

A total of 109 patients undergoing selective non-emergency, non-cardiothoracic or non-neurosurgical surgery requiring tracheal intubation for general anesthesia were enrolled. Ten patients per group were assigned to ciprofol-0.3, 0.4 and 0.5 mg/kg, and propofol-2.0 or 2.5 mg/kg groups, respectively to receive an intravenous bolus dose. An additional 20 patients were enrolled in the ciprofol-0.3, 0.5 or propofol-2.0 mg/kg groups. The primary outcome was the success rate of induction defined as a Modified Observer’s Assessment of Alertness/Sedation (MOAA/S) ≤ 1 after the initial bolus dose. The secondary outcomes included the time to reach MOAA/S ≤ 1, the time to loss of the eyelash reflex, the incidences and severity of adverse events (AEs).

**Results:**

The success rates were 100% for all 5 groups. The mean time to MOAA/S ≤ 1 and the time to loss of the eyelash reflex were not different among the 5 groups, regardless of whether a top-up dose was needed. There were no significant differences in the incidences and severity of AEs in the dose ranges investigated of ciprofol vs. propofol.

**Conclusion:**

The efficacy and safety of a single bolus dose of ciprofol-0.5 mg/kg for the general anesthesia induction in selective surgery patients was comparable to that of propofol-2.0 mg/kg.

**Trial registration:**

Clinicaltrials.gov, NCT03698617, retrospectively registered.

**Supplementary Information:**

The online version contains supplementary material available at 10.1007/s11096-022-01529-x.

## Impact statements

- Propofol is commonly used for procedural sedation, but since it has the disadvantage of a narrow therapeutic index, alternative sedation drugs with improved drug dosing properties are desirable.
- Ciprofol is a novel drug for procedural sedation and general anesthesia with a potency about 4–5 times higher than propofol, which reduces the necessary amount of active drug required to induce sedation compared to propofol.
- In this phase 2a clinical trial, 0.5 mg/kg ciprofol had similar outcomes to 2.0 mg propofol but a future randomized study is warranted.

## Introduction

Propofol is commonly used for procedural sedation [1], induction of general anesthesia [2], total intravenous anesthesia [3] and sedation in intensive care units (ICU) [4]. The efficacy and safety of sedation produced by propofol has been well established [5–7]. Known side effects associated with propofol use mainly include apnea, cardiovascular depression, pain on injection, and rarely but more seriously propofol infusion syndrome, which remains a problem even with newer formulations [8, 9]. Thus, there is a clinical need for a new sedative anesthetic drug with similar desirable effects to propofol, but with fewer adverse events (AEs).

Ciprofol emulsion (HSK3486), a 2, 6-disubstituted phenol derivative [10], has been newly developed for procedural sedation and general anesthesia. It is a highly selective gamma-aminobutyric acid-A (GABA_A_)-receptor agonist [11], that is similar to propofol [12, 13]. Ciprofol has favorable pharmacokinetic (PK) characteristics, pharmacodynamic responses and safety at a 0.4 mg/kg study dose [14]. Our phase 1 clinical trial on ciprofol (dose range 0.15–0.9 mg/kg) was conducted to assess the appropriate dose for sedation, safety, tolerance and its PK properties in healthy subjects [15]. The dose normalized PK parameters C_max_, t_max_, t_1/2_, k_z_ and MRT were similar, while CL, V_d_ and V_ss_ were statistically significantly lower after patients received a 4 h-infusion of ciprofol vs. propofol [16]. The previous phase 2a + 2b trials conducted in Chinese patients undergoing colonoscopy showed that ciprofol was well tolerated at doses ranging from 0.1 to 0.5 mg/kg, and that ciprofol 0.4–0.5 mg/kg had an equivalent sedation/anesthesia profile to propofol-2.0 mg/kg during colonoscopy [15]. In addition to the comparable anesthesia/sedation profiles with propofol, the significant advantage for ciprofol was the lower incidences of pain on injection and the smaller amount of lipid emulsion input [15, 17, 18].

We assumed that ciprofol would have a similar success rate to propofol for the induction of general anesthesia and here we also provide guidance for its the clinical applications.

### Aim

Based on previous phase 2 results, in this phase 2 study, we aimed to: (1) determine the equally potent dose of ciprofol compared with propofol as an induction agent for general anesthesia in patients undergoing selective surgery; and (2) to assess its safety.

### Ethics approval

The study was approved by the Institutional Review Board (IRB) of Peking University First Hospital (Approval No. 2016-46; December 28th, 2016), West China Hospital, Sichuan University (Approval No. 2017-32; January 15th, 2018) and all other participating medical centers. Prior written informed consent was obtained from all participants in the trial.

## Method

### Study design and patients

This was a phase 2a, 7-center, open-labeled, non-randomized and positive controlled clinical trial. The study consisted of two parts: part 1, dose escalation and part 2, dose expansion. The trial was registered at clinicaltrials.gov (NCT03698617).

The inclusion criteria for participants were: 18–65 years old; non-pregnant and non-lactating if female; body mass index (BMI) 19–30 kg/m^2^; American Society of Anesthesiologists (ASA) physical status I-II; and scheduled for selective, non-cardiothoracic or non-neurosurgical surgery that required tracheal intubation for general anesthesia with estimated operation times of 1–3 h. Exclusion criteria were: a history of AEs during previous anesthesia or were allergic to propofol or other anesthetic drugs; individuals who had participated in any clinical trial in the previous 3 months; or had received propofol or opioids within 1 month or general anesthesia within 2 weeks of the trial commencing. More detailed information about the criteria are given in Supplementary File 1.

### Part I: Dose escalation

In a previous phase 1 study, the results indicated that ciprofol 0.4 mg/kg, administered as a single i.v. bolus dose, was suitable for inducing general anesthesia in healthy adults. Therefore, in this phase 2a study, we chose 3 doses of ciprofol (0.3, 0.4, 0.5 mg/kg) and 2 doses of propofol (2.0, 2.5 mg/kg). The plan was to enroll 10 patients in each group, with 0.4 mg/kg ciprofol selected as the initial escalation dosage, based on the results of a phase 1 study. If 10 patients in the ciprofol 0.4 mg/kg all successfully achieved 100% induction of anesthesia with a single bolus injection, the next step was administration of a single dose of 0.3 mg/kg for induction, with a dose reduction of 0.1 mg/kg for the 10 patients (ciprofol 0.3 mg/kg group). If one or more of the 10 patients who received a dose of 0.4 mg/kg failed to achieve satisfactory anesthesia, a dose of 0.5 mg/kg was given to another 10 patients, with an increment in the dose of 0.1 mg/kg (ciprofol 0.5 mg/kg group). It should be noted that the investigator and sponsor needed to decide whether patients could receive the next dose, after they had evaluated the safety and efficacy outcomes of the previous dose. For propofol, doses of 2.0 mg/kg and 2.5 mg/kg were arbitrarily chosen as guided by a previous study [19, 20]. In the present trial, 2.0–2.5 mg/kg propofol was selected as the positive control dosage range.

Finally, 9, 8 and 8 patients were scheduled to be enrolled in the 3 ciprofol groups and 10 in each propofol group.

#### For top-up doses and numbers selection

The sedation level was assessed using the Modified Observer’s Assessment of Alertness/Sedation scores (MOAA/S) [21] prior to the initiation of induction, and then every 1 min after the administration of ciprofol or propofol. The success of induction was defined as a MOAA/S score ≤ 1, 2 min after the start of ciprofol or propofol administration. Otherwise, an additional top-up dose (50% of the initial dose) was administered. The second top-up dose was given if the MOAA/S score was still > 1, 3 min after the start of ciprofol or propofol administration. If the MOAA/S score remained at > 1, 5 min after the initial i.v. bolus, it was defined as induction failure and the care team was allowed to manage the sedation as they wished, usually by giving an additional dose of propofol.

### Part II: Dose expansion

Upon completion of the part I study, a part 2 study was performed. Based on the results of the 5 groups (n = 10 for each group the study started with), an additional 20 patients were planned to be enrolled in each of the ciprofol-0.3, 0.5 mg/kg and propofol-2.0 mg/kg groups, respectively.

### Study procedure

A participant’s intravenous cannula was inserted into a vein in the right arm (dose escalation group) or a vein catheter (dose expansion group) for administration or top-up of the study drug. Only patients in the ciprofol group required an open vein in the left arm for blood collection for PK analysis. A simultaneous arterial catheter was only placed in the radial artery of 4 patients in the 0.4 mg/kg ciprofol group for arterial blood collection.

Standard monitoring was applied including a 12-lead ECG, pulse oxygen saturation (SpO_2_), non-invasive blood pressure measurements, temperature and end-tidal carbon dioxide (EtCO_2_). Midazolam 0.04 mg/kg (15 s, i.v.) and sufentanil 0.3 µg/kg (30 s, i.v.) were given once pre-oxygenation started. Then ciprofol or propofol was administered as a single bolus i.v. injection approximately 2 min after midazolam and sufentanil were given. The pre-prepared ciprofol or propofol dose was administered i.v. as a single bolus dose over approximately 30 s.

### End points

The primary end-point was the success rate of induction of general anesthesia produced by the initial bolus dose, i.e. the percentage of patients who achieved successful induction after the initial bolus dose, without requiring a top-up dose.

The secondary end-points were: (1) the time to reach a MOAA/S ≤ 1, defined as the time from the first administration of ciprofol or propofol to the first occurrence of MOAA/S ≤ 1; (2) the time for loss of the eyelash reflex, defined as the time from the first administration of ciprofol or propofol to disappearance of the eyelash reflex; (3) emergence time, defined as the time from the completion of surgery to the first occurrence of MOAA/S = 5; (4) number of top-up doses; (5) incidence and severity of AEs during the whole study, number of corresponding treatments, and the accumulated doses of rescue medication for AEs within 15 min after the initiation of induction.

### Definition of AEs and grading

The definition and grading of key AEs and treatment measures are listed in Supplementary File 2, as well as the definition of serious AEs. In the case of AEs not described in Supplementary File 2 or the National Institutes of Health-Common Terminology Criteria for Adverse Events (CTCAE ver. 4.0) guidelines [22], their severity was graded as follows: grade 1 (mild), asymptomatic or mild, no treatment required; grade 2 (moderate), required minor/local or noninvasive treatment; grade 3 (severe), not immediately life-threatening, but could lead to hospitalization or extended hospitalization, or disability, or affect a person’s daily life style; grade 4 (life-threatening), required emergency treatment; grade 5 (death), AEs related.

The trial termination criteria were:

1. *Upper limit of ciprofol dose*: Escalation reached a dose as a single bolus at which the success rate of induction was the same as for propofol at a dose of 2.0 or 2.5 mg/kg.
2. *Lower limit of ciprofol dose*: Escalation reached a dose as a single bolus at which the success rate of induction was 90%, or a failure occurred.
3. More than 50% of the patients in any of the ciprofol groups had severe AEs (Supplementary File 2), or other AEs ≥ grade 3 according to CTC-AE 4.0.
4. One or more serious AEs (Supplementary File 2) in any of the ciprofol groups that occurred during the dose escalation study.

### Measurements

The pain associated with a ciprofol or propofol injection was assessed approximately 15 s after initiation of the bolus injection. MOAA/S scores were recorded every 1 min and the eyelash reflex every 30 s.

Hemodynamic parameters including HR, SBP, DBP and mean arterial blood pressure (MAP) were recorded at intervals of 1 min within the first 10 min, and at intervals of 5 min thereafter. SpO_2_, EtCO_2_ and ECG were continuously monitored throughout the procedure. The area under the curves (AUCs) of SBP, DBP, MAP and HR 20% below baseline were analyzed to assess the effect of over sedation on hemodynamic stability. A similar approach was used to analyze the inadequacy of sedation in terms of the patients’ responses to intubation, an AUC 20% above the baseline of blood pressure and HR within 15 min of initiation of induction. All AEs and medications given were carefully documented throughout the entire procedure.

#### Emergence time

Once surgery was completed, the MOAA/S score was assessed every 2 min until patients’ MOAA/S scores reached 5.

### Drugs

#### Investigational drugs

Ciprofol emulsion (20 mL: 200 mg in part 1, and 20 mL: 50 mg in part 2 (Liaoning Haisco Pharmaceutical Group Co., Ltd., China) and Diprivan® (20 mL: 200 mg, AstraZeneca, UK).

Midazolam (Jiangsu Nhwa Pharmaceutical Co., Ltd., China), sufentanil (Yichang Humanwell Pharmaceutical Co., Ltd., China) and rocuronium bromide (Esmeron, N.V. Organon, The Netherlands) were given in combination during the induction of general anesthesia.

### Statistical analysis

IBM SPSS (ver. 25) was used for all statistical analyses. The demographic characteristics were analyzed using analysis of variance (ANOVA), chi-square, Fisher or Kruskal–Wallis tests. The primary outcome was also analyzed using Wilson’s and/or Fisher tests. Time to MOAA/S ≤ 1 and loss of the eyelash reflex were evaluated using the Kruskal–Wallis test, followed by Student's *t*-test for multiple comparisons. Time to emergence was analyzed using one-way ANOVA and most AEs using Fisher’s test. The AUCs of vital signs was evaluated using the Kruskal–Wallis test. Data are expressed as the mean ± SD or as percentages. Results reported as significant are based on a *P*-value < 0.05.

The sample size calculation was based on the data from our previous clinical experience. Assuming that the success rate of induction with propofol injection was 100% and the difference in the success rate of induction with a ciprofol equally potent dose was not > 3%, a sample size of 23 patients in each group would produce a two-sided 95% confidence interval (CI) of − 0.1 to 0.1. Considering that the dropout rate would be about 25%, the sample size was rounded to 30 patients in the dose escalation + expansion study. Therefore, we started with 10 patients in each group in the part 1 dose escalation and planned to add another 20 patients in the part 2 dose expansion.

## Results

### Demographic characteristics of patients in the dose escalation and dose expansion groups

**Fig. 1:**
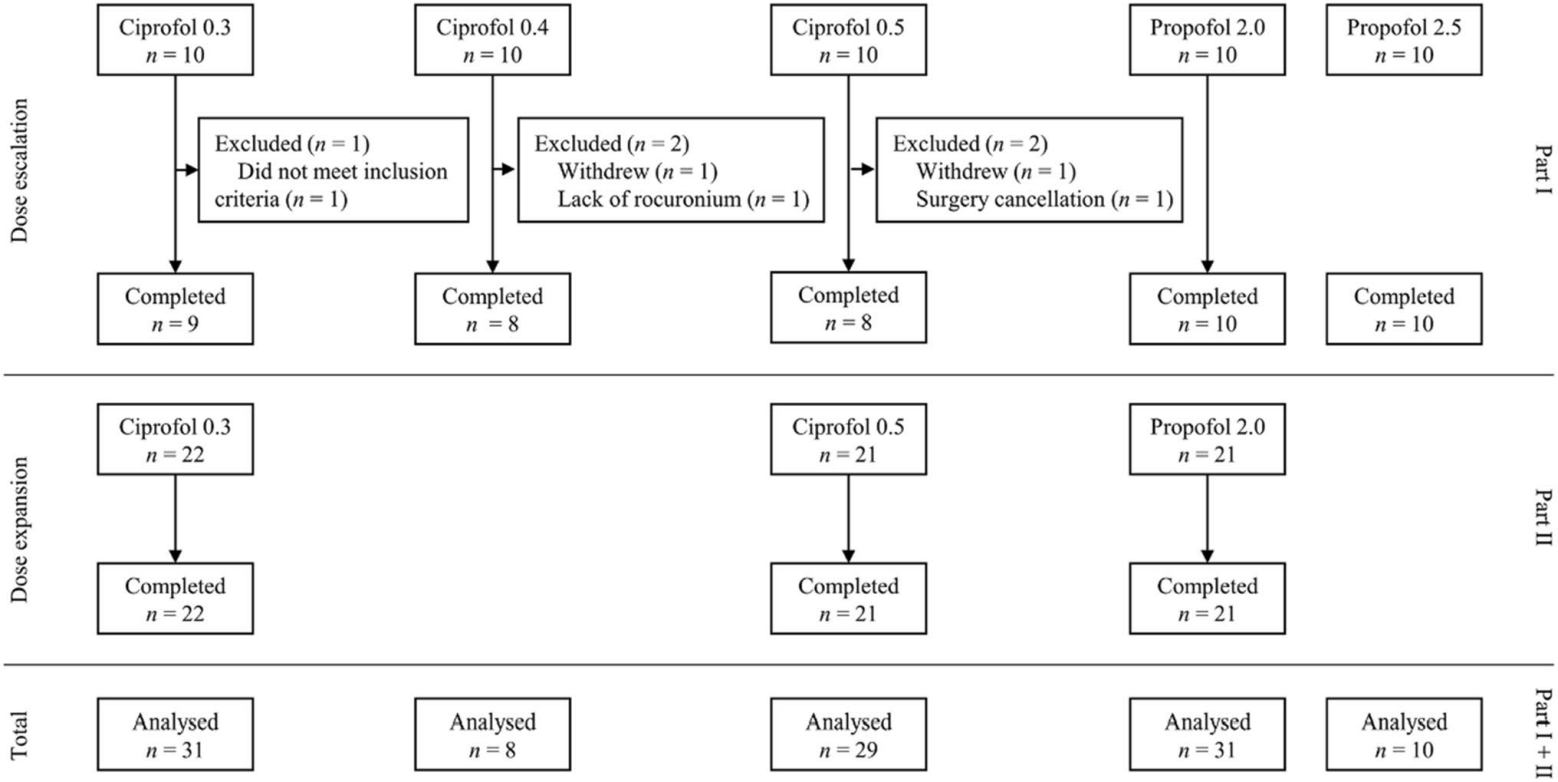
Flow chart of patient enrollment

From December 2016 to July 2018, a total of 109 patients for inclusion in both part 1 and part 2 were enrolled and the flow chart is shown in Fig. 1. In the ciprofol groups, a total of 5 patients were excluded in part 1. Two patients withdrew after signing their consents before induction; 1 was excluded due to a baseline electrocardiogram abnormality; 1 due to the unavailability of rocuronium bromide data; and another patient due to cancellation of their surgery. Finally, there were 22, 21 and 21 patients enrolled in the ciprofol-0.3, 0.5 mg/kg and propofol-2.0 mg/kg groups, respectively, in the part 2 dose expansion. The patients involved in the present study mainly required abdominal or urinary surgery, thyroidectomy, otorhinolaryngology, plastic or gynecological surgery, or other types. The demographic characteristics of the enrolled patients are shown in Supplementary Table 1. There was a significant difference in the ASA status distribution (*P* = 0.036) but there was no significant difference among the 5 groups with regard to the other variables investigated.

### Efficacy

#### Primary outcome

The success rates of anesthesia induction in patients who received one initial bolus dose of ciprofol-0.5 mg/kg, propofol-2.0 and 2.5 mg/kg were all 100%. However, 1 patient in the ciprofol-0.4 mg/kg group (part I) and one in the ciprofol-0.3 mg/kg group (part II) required one top-up dose and then achieved successful induction. No patient failed the induction and no patient received more than one top-up dose (Table 1).

**Table 1:** Efficacy of sedation in all patients with ciprofol or propofol including dose escalation and dose expansion

Induction drug	Ciprofol (mg/kg)			*P*-value*	Propofol (mg/kg)		*P*-value (Ciprofol vs propofol)
	0.3 (n = 31)	0.4 (n = 8)	0.5 (n = 29)		2.0 (n = 31)	2.5 (n = 10)	
Patients who achieved successful induction with the initial bolus, n	30	7	29	1.000	31	10	1.000
*Success rate, n (%)*							
Part I: dose escalation	9 (100)	7 (87.5)	8 (100)	1.000	10 (100)	10 (100)	
Part II: dose expansion	21(95.5)		21 (100)	1.000	21 (100)		
*Time to MOAA/S score ≤ 1, n*							
At end of the 1 min	30	6*	29	0.035	30	10	0.135
At end of the 2 min	0	1	0	0.118	1	0	0.135
*Time to loss of the eyelash reflex (sec), mean ± SD*	42.6 ± 16.2	48.0 ± 13.8	39.6 ± 15.0	0.382	43.8 ± 16.2	42.0 ± 15.6	0.724
Part I: dose escalation	43.6 ± 16.1	51.3 ± 19.8	41.3 ± 15.5	0.485	47.5 ± 17.0	42.0 ± 15.5	
Part II: dose expansion	45.6 ± 21.2		39.3 ± 15.3	0.272	42.4 ± 16.3		
Duration of successful induction of anesthesia (min), mean ± SD*							
Part I: dose escalation	1.0 ± 0.0	1.4 ± 0.8	1.0 ± 0.0	0.142	0.9 ± 0.6	1.0 ± 0.0	
Part II: dose expansion	0.9 ± 0.3		0.7 ± 0.3	0.035	0.7 ± 0.3		
Emergence time (min), mean ± SD	14.3 ± 8.2	10.7 ± 5.4	15.5 ± 10.3	0.409	15.6 ± 10.1	14.4 ± 10.5	0.779
Patients with the initial bolus plus once top-up, n	31	8	29	1.000	31	10	1.000
Success induction rate, n (%)	31 (100.0)	8 (100.0)	29 (100.0)	1.000	31 (100.0)	10 (100.0)	1.000
Time to loss of the eyelash reflex (sec), mean ± SD	45.0 ± 19.8	53.4 ± 19.8	39.6 ± 15.0	0.141	43.8 ± 16.2	42.0 ± 15.6	0.491
Emergence time (min), mean ± SD	13.9 ± 8.3	9.6 ± 5.9	15.5 ± 10.3	0.265	15.6 ± 10.1	14.4 ± 10.5	0.570

Successful time of anesthesia induction = the time when MOAA/S ≤ 1 occurred—the time when the study drug was first administered; Time to loss of the eyelash reflex = eyelash reflex disappearance time—the time when the study drug was first administered; Emergence time was defined as the period from surgery completion to a MOAA/S score = 5

*P < 0.05 when compared between ciprofol-0.4 and 0.5 mg/kg groups

#### Secondary outcomes

All of the patients who received an initial bolus reached MOAA/S scores ≤ 1 at the end of the first min of administration except for 2 patients (1 in the 0.4 mg/kg ciprofol group and the other in the 2.0 mg/kg propofol group), who achieved MOAA/S scores ≤ 1 at the end of the second min of drug administration).

As shown in Table 1, the time to loss of the eyelash reflex was shortest in the 0.5 mg/kg ciprofol group (39.6 ± 15.0 s); duration of successful induction of anesthesia in the 0.4 mg/kg ciprofol group was 1.4 ± 0.8 min, which seemed longer than in any of the other ciprofol or propofol groups, and the emergence time in all groups clearly showed that the period from surgery completion to a MOAA/S score = 5 in the 0.4 mg/kg ciprofol was the shortest (10.7 ± 5.4 min) (all *P* > 0.05).

Furthermore, no significant differences were found between the time to loss of the eyelash reflex and the time to emergence after the operation among the 5 groups, even when the patients who required one top-up dose were included in the analysis (all *P* > 0.05).

### Safety

No patient in either the part I or II trials had AEs severe enough to trigger trial termination. All the AEs detected are listed in Supplementary Table 2. There were no significant differences in the incidence of total AEs among the 5 groups, or in their severity levels: mild (71.6%), moderate (55.9%), severe (21.1%), and serious (0.9%) (Table 2). One serious AE occurred in the propofol 2.5 mg/kg group as the patient became pregnant 1 month after the trial. Her pregnancy ended with elective abortion at 25 weeks due to a fetal sex chromosome abnormality. The most common AEs during the whole study period included hypotension (36.7%), hypertension (55.0%), sinus bradycardia (43.1%), sinus tachycardia (15.6%), QTcF interval prolongation (27.5%), nausea (11.0%) and/or vomiting (10.1%). One patient in the ciprofol 0.3 mg/kg group exhibited urticaria 10 min after ciprofol administration and was treated successfully with 10 mg of dexamethasone.

**Table 2:** Summary of AEs that occurred during the entire study period

Induction drug	Ciprofol (mg/kg)			Propofol (mg/kg)		Total (n = 109)
	0.3 (n = 31)	0.4 (n = 8)	0.5 (n = 29)	2.0 (n = 31)	2.5 (n = 10)	
Total number of AEs, n	217/31	33/8	161/29	159/30	73/10	642/108
*Severity of AEs, n (%)*						
Mild	22 (70.9)	4 (50.0)	25 (86.2)	25 (80.6)	2 (20.0)	78 (71.6)
Moderate	20 (64.5)	2 (25.0)	17 (58.6)	18 (58.1)	4 (40.0)	61 (55.9)
Severe	4 (12.9)	2 (25.0)	8 (27.6)	5 (16.1)	4 (40.0)	23 (21.1)
Serious	0	0	0	0	1 (10.0)	1 (0.9)
Hypotension	8 (25.8)	2 (25.0)	12 (41.4)	15 (48.4)	3 (30.0)	40 (36.7)
Hypertension	21 (67.7)	4 (50.0)	13 (44.8)	14 (45.1)	8 (80.0)	60 (55.0)
Sinus bradycardia	11 (35.5)	4 (50.0)	13 (44.8)	17 (54.8)	2 (20.0)	47 (43.1)
Sinus tachycardia	6 (19.4)	1 (12.5)	3 (10.3)	4 (12.9)	3 (30.0)	17 (15.6)
Ventricular extrasystole	1 (3.2)	0	1 (3.5)	0	0	2 (1.8)
QT interval prolongation	8 (25.8)	2 (25.0)	9 (31.0)	8 (25.8)	3 (30.0)	30 (27.5)
ECG ST segment depression	1 (3.2)	0	1 (3.5)	1 (3.2)	0	3 (2.8)
Oxygen desaturation	5 (16.1)	1 (12.5)	3 (10.3)	4 (12.9)	1 (10.0)	14 (12.8)
Injection pain	2 (66.7)	0	4 (13.8)	7 (22.6)	1 (10.0)	14 (12.8)
Vomiting	4 (12.9)	0	3 (10.3)	3 (9.7)	1 (10.0)	11 (10.1)
Nausea	2 (6.5)	0	3 (10.3)	4 (12.9)	3 (30.0)	12 (11.0)
Urticaria	0	1 (12.5)	0	0	0	1 (0.9)
Dizziness	4 (12.9)	0	2 (6.9)	1 (3.2)	3 (30.0)	10 (9.2)
Headache	2 (6.5)	0	0	1 (3.2)	1 (10.0)	4 (3.7)
Dyskinesia	2 (6.5)	0	0	0	0	2 (1.8)
Fever	2 (6.5)	0	0	0	0	2 (1.8)
Chill	2 (6.5)	0	0	1 (3.2)	1 (10.0)	5 (4.6)
Insomnia	0	0	0	0	2 (20.0)	2 (1.8)

**Fig. 2:**
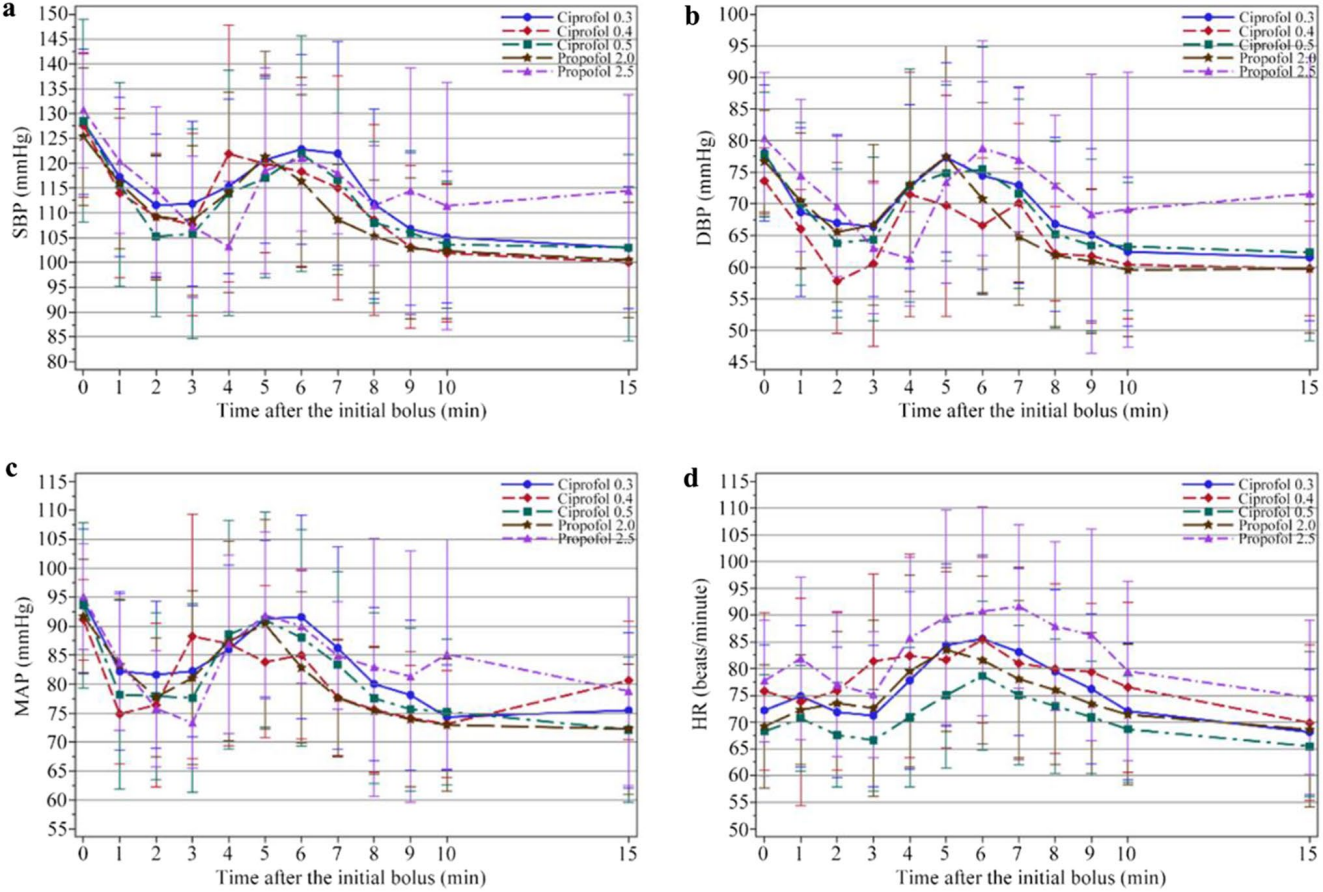
Heart rate and blood pressure of patients within 15 min of receiving the initial bolus of ciprofol vs. propofol. **a** Systolic blood pressure (SBP), **b** diastolic blood pressure (DBP), **c** mean arterial blood pressure (MAP), **d** heart rate (HR), beats/min

**Fig. 3:**
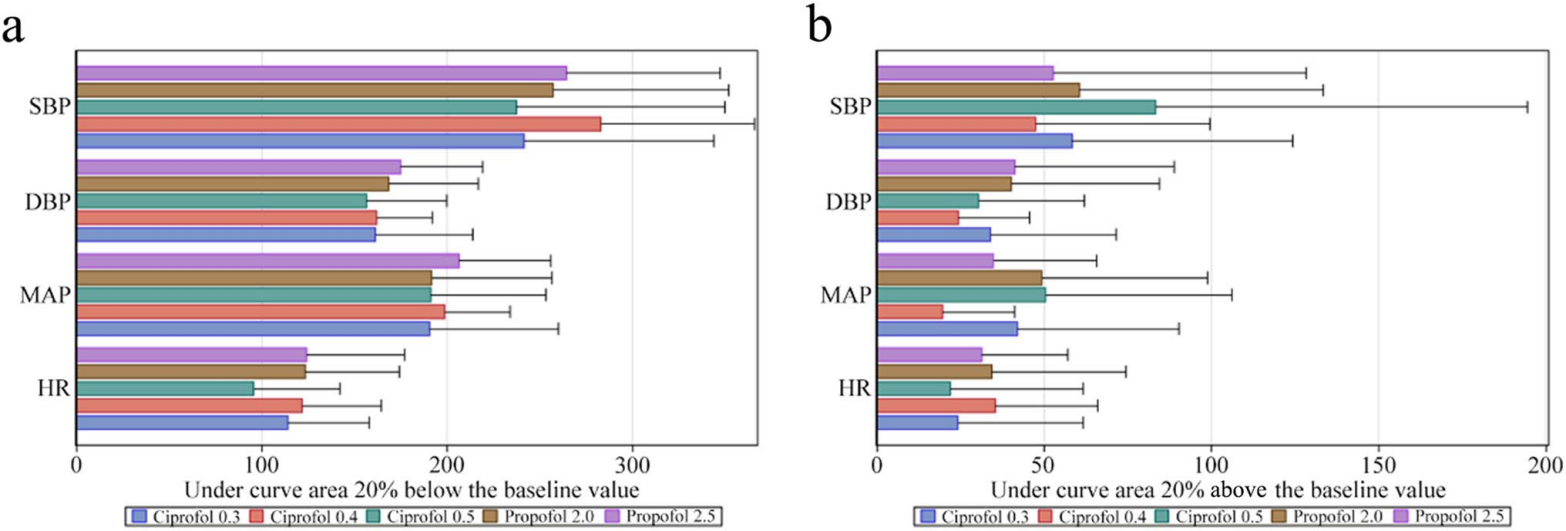
**a** Area under the curve (AUC) 20% below the baseline value, and **b** AUC 20% above the baseline value of each HR and blood pressure variable multiplied by time (min). Above and below AUCs were analyzed using Kruskal–Wallis H tests, and there were no significant differences among the 5 groups for each variable investigated

Figure 2 shows SBP, DBP, MAP and HR within 15 min of ciprofol or propofol administration for all 5 groups. There were no significant differences in AUCs, either below or above the baseline values of hemodynamics in the 5 groups (Fig. 3). No significant differences were found in the incidence of hypotension, hypertension, sinus bradycardia or the frequency of their corresponding treatments (Table 3). The rescue drugs included ephedrine 3 mg i.v., metaraminol 0.3 mg i.v. or methoxamine 1 mg i.v. for the treatment of hypotension, and atropine 0.3 or 0.25 mg i.v. for the treatment of sinus bradycardia. There were 4 cases of QTcF interval prolongation; 2 of them occurred in the ciprofol 0.4 mg/kg group and 2 in the propofol 2.5 mg/kg group. The QTcF interval prolongation in all 4 cases was transient and returned to a normal range without intervention. No tachycardia was observed within 15 min of induction and only 15.6% of patients experienced tachycardia during the entire study.

**Table 3:** Incidence of cardiovascular AEs and the corresponding rescue drugs within 15 min from the initiation of induction

	Hypotension		Hypertension		Sinus bradycardia		QTcF interval prolongation	
	n (%)	Treatment, n	n (%)	Treatment, n	n (%)	Treatment, n	n (%)	Treatment, n
Ciprofol-0.3 mg/kg (n = 31)	4 (12.9)	4	0	–	1 (3.2)	1	0	–
Ciprofol-0.4 mg/kg (n = 8)	0	–	0	–	1 (12.5)	0	2 (25.0)	0
Ciprofol-0.5 mg/kg (n = 29)	6 (13.8)	6	1 (3.4)	0	2 (6.9)	2	0	–
Propofol-2.0 mg/kg (n = 31)	6 (19.4)	5	0	–	0	–	0	–
Propofol-2.5 mg/kg (n = 10)	1 (10.0)	1	0	–	0	–	2 (20.0)	0

Hypertension was defined as systolic pressure < 90 mmHg; Hypertension systolic pressure > 139 mmHg; Bradycardia was defined as HR < 50 beats/minute; QTcF interval prolongation was defined as QTcF interval ≥ 450 ms (corrected by Friderica's formula). No patient presented with tachycardia (HR > 100 beats/minute)

## Discussion

Propofol has been reported to have a narrow therapeutic index and the guidelines recommend usage only by experienced anesthesiologists, with close monitoring and individual dose adjustments [23, 24]. An alternative strategy has been developed as a balanced sedation protocol using midazolam in combination with a minimized dose of propofol for prescription by a non-anesthesiologist. However, a study noted that in clinical practice, propofol is commonly administered at doses of 1.8 to 2.2 mg/kg above the recommended doses required for the induction of anesthesia, with an incidence rate of 26% propofol-related intraoperative hypotension especially in elderly patients [25–27]. In previous animal experiments, the therapeutic index of ciprofol was found to be 1.5 times that of propofol [11], which might lead to simplified clinical applications in real clinical settings especially for elderly patients.

The main finding of the present study was that the success rates of induction with ciprofol-0.5 mg/kg as an i.v. bolus was comparable to that of propofol-2.0 mg/kg. When lower doses of ciprofol (0.3 or 0.4 mg/kg) were given, a top-up dose was occasionally needed after the initial bolus injection to achieve a 100% success rate of induction. The results also showed that patients in ciprofol-0.5 mg/kg group had the shortest time of loss of the eyelash reflex and that the 0.4 mg/kg group had the longest duration for successful induction and the shortest emergence times. In terms of safety, there were no significant differences among the ciprofol and propofol groups in the incidence of AEs. Vital signs were relatively stable during induction of general anesthesia and even during intubation, and no significant differences were found in term of the accumulated dose of the rescue drugs used to treat AEs. Considering the efficacy and safety endpoints, ciprofol 0.5 mg/kg for induction of general anesthesia was as effective and safe as propofol at a dose of 2.0 mg/kg. No difference in hemodynamic stability during the induction period among the 5 groups was due to prompt treatment with rescue drugs.

There is another potential advantage of ciprofol, namely less injection pain on administration compared to propofol. This conjecture is based on the fact that the injection pain produced by propofol is attributable to the absolute drug concentration in the aquatic phase [28, 29]. A concentration of ciprofol of 50 mg/20 mL (vs. propofol at 200 mg/20 mL) should dramatically reduce the concentration in the aquatic phase and therefore the degree of injection pain [10]. Of course, this conjecture will be tested in our phase 3 study.

There were a number of limitations to the study. First, patients were not randomly assigned to groups and a future randomized study will be conducted. In addition, selection bias might have existed as the differences in ASA statuses between the 5 groups were significant (Supplementary Table 1). Second, the administration of the premedication midazolam and sufentanil, but the study design was intended to reflect real clinical practice as much as possible. It should however be noted that the use of a combination of midazolam and sufentanil as premedication has been standard practice in clinical anesthesia for a number of years [30, 31]. Nevertheless, no difference was found between them and the non-protocolization of anesthesia maintenance should not have had an effect on the conclusions drawn.

## Conclusion

The efficacy and safety of a single i.v. bolus dose of 0.5 mg/kg ciprofol for the induction of general anesthesia in patients undergoing selective surgery was comparable to propofol administered at a dose of 2.0 mg/kg. Lower doses of ciprofol, 0.3 mg/kg or 0.4 mg/kg, could also achieve the same efficacy but occasionally an additional top-up dose was required.

## Supplementary Information

Below is the link to the electronic supplementary material.

Supplementary file1 (DOCX 45 kb)
