# Supplementary material for: Efficacy and safety of ciprofol versus propofol for the induction of anesthesia in adult patients: a multicenter phase 2a clinical trial
Source: Int J Clin Pharm. 2023 Jan 21;45(2):473–82. doi: 10.1007/s11096-022-01529-x (PMC10147789; doi:10.1007/s11096-022-01529-x)
Supplement: Supplementary file 1 — Supplementary file1 (DOCX 45 kb) [file 11096_2022_1529_MOESM1_ESM.docx]

**Supplementary File 1. Inclusion and exclusion criteria**

***Inclusion criteria included***: age 18-65 years, non-pregnant and non-lactating if a patient was a female, BMI 19-30 kg/m^2^ and ASA physical status I–II, scheduled for selective, non-cardiothoracic or non-neurosurgical surgery that required tracheal intubation for general anaesthesia with estimated operation times of 1-3 h. The patients who can understand the procedures and methods of this study, is willing to strictly follow the clinical trial protocol to complete the study, and signed the informed consent.

***Exclusion criteria included***: 1) a history of adverse events during previous anaesthesia or were allergic to propofol or other anesthetic drugs; 2) those who had participated in any clinical trial in the previous 3 months, received propofol or opioids within 1 month, or had general anaesthesia within 2 weeks of the trial commencing; 3) an electrocardiogram (ECG) QTcF interval ≥ 450 ms (corrected by Fridericia's formula); 4) base line heart rate (HR) < 50 or > 100 bpm; systolic blood pressure (SBP) > 160 mmHg or < 90mmHg, or diastolic blood pressure (DBP) > 100 mmHg or < 60 mmHg; 5) abnormal laboratory results consisting of any of the following were exclusion criteria: AST or ALT ≥ 2 × ULN (upper limit of normal); TBIL ≥ 1.5 × ULN; Hb ≤ 90 g dk^-1^; ANC ≤ 1.5 × 10^9^ L^-1^; PLT ≤ 80 × 10^9^ L^-1^; serum creatinine ≥ 1.5 × ULN; and 6) patients who intended to get pregnant within 1 month prior to the trial or 1 month after the trial.

**Supplementary File 2. Severity classification and treatment measures of key adverse events (AEs), and the definition of serious AEs**

**1. Severity classification and treatment measures of key adverse events**

- **Hypotension**

1. Mild: Systolic blood pressure 80-89 mmHg and the duration time ≥ 5 min.

*Treatment*: Careful observation without medication.

1. Moderate: Systolic blood pressure 60-79 mmHg and the duration time ≥ 5 min after treatment.

*Treatment*: Hydroxyamine 0.1 mg, iv.

1. Severe: Systolic blood pressure 40-59 mmHg and the duration time ≥ 5min after treatment.

*Treatment*: Hydroxyamine 0.2-0.3 mg or ephedrine 6 mg, iv; these measures repeated if necessary.

1. Serious: Systolic blood pressure < 40 mmHg, and after treatment the result of ≥ 3 times in 5 consecutive blood pressure measurements was still less than 40 mmHg.

*Emergency treatment*: Hydroxyamine 0.6-1 mg, or ephedrine 12 mg iv, or a continuous dopamine infusion at a rate of 1-20 μg/kg/min if necessary. If not relieved after 3 minutes, repeat these measures immediately. If not relieved after 3 minutes, epinephrine may also be used one or more times.

*Cardiopulmonary resuscitation*: CPR if necessary.

1. Death

- **Hypertension**

1. Mild: Systolic blood pressure 140-149 mmHg or diastolic pressure 140-149 mmHg and a duration time ≥ 5min.

*Treatment*: Careful observation without medical treatment.

1. Moderate: Systolic blood pressure 150-159 mmHg or diastolic pressure 95-102 mmHg and a duration time ≥ 5 min after treatment.

*Treatment*: Nicardipine hydrochloride 0.2 mg iv.

1. Severe: Systolic blood pressure 160-180 mmHg or diastolic pressure 103-110 mmHg, and the duration time ≥ 5min after treatment.

*Treatment*: Nicardipine hydrochloride 0.2 mg iv or urapidil hydrochloride 12.5 mg iv; these measures repeated if necessary.

1. Serious: Systolic blood pressure > 180 mmHg or diastolic pressure > 110 mmHg, and after treatment the result of ≥ 3 times in 5 consecutive blood pressure measurements was SBP > 180 mmHg or DBP > 110 mmHg.

*E*m*ergency treatment*: Nicardipine hydrochloride 0.4 mg or urapidil hydrochloride 25 mg iv slowly; repeat these measures and continue nitroglycerin infusion at rate 0.1-1.0 μ g/kg/ min if necessary.

*Cardiopulmonary resuscitation*: CPR if necessary.

1. Death

- **Sinus bradycardia**

1. Mild: Heart rate 50-55 bpm and duration time ≥ 5min.

*Treatment*: Careful observation without medical treatment.

1. Moderate: Heart rate 40-49 bpm, and the duration time ≥ 5min after treatment.

*Treatment*: Atropine 0.3 mg iv.

1. Severe: Heart rate 35-39 bpm, and the duration time ≥ 5min after treatment.

*Treatment*: Atropine 0.3 mg iv, repeat these measures if necessary.

1. Serious: Heart rate < 35 bmp, and after treatment the result of ≥ 3 times in 5 consecutive the heart rate was still less than 35 bmp.

*Emergency treatment*: Dopamine 1 mg iv, repeat these measures if necessary, and continue dopamine infusion at rate 1-20 μg/kg/min and/or adrenaline 1 mg iv, repeat every 3-5 min if necessary.

*Cardiopulmonary resuscitation*: CPR if necessary.

1. Death

- **Sinus tachycardia**

1. Mild: Heart rate 101-129 bpm and duration time ≥ 5min.

*Treatment*: Careful observation without medical treatment.

1. Moderate: Heart rate 130-149 bpm, and the duration time ≥ 5min after treatment.

*Treatment*: Esmolol 20 mg iv.

1. Severe: Heart rate 150-180 bpm, and the duration time ≥ 5 min after treatment.

*Treatment*: Esmolol 20 mg iv, repeat these measures if necessary.

1. Serious: Heart rate > 180 bmp, and after treatment the result of ≥ 3 times in 5 consecutive the heart rate was still more than 180 bmp.

*Emergency treatment*: Esmolol 40 mg iv, repeat these measures and prepare for cardioversion if necessary.

*Cardiopulmonary resuscitation*: CPR if required.

1. Death

- **Hypoxia**

1. Mild: SpO_2_ 90-94%, and duration time ≥ 5min.

*Treatment*: Careful observation without medical treatment.

1. Moderate: SpO_2_ 80-89%, and the duration time ≥ 5min after treatment;

*Treatment*: Increase oxygen flow rate to deal with the situation of hypoxia. If there is an obstruction of upper respiratory tract, a nasal or oral airway should be placed. If bronchospasm occurs, adrenaline 5-10 μg should be given intravenously; if laryngospasm occurs, propofol 2 mg/kg should be given intravenously.

1. Severe: SpO_2_ 70-79%, and the duration time ≥ 5min after treatment.

*Treatment*: Positive pressure ventilation with mask immediately to deal with the situation of hypoxia. If there is an obstruction of upper respiratory tract, a nasal or oral airway should be placed. If bronchospasm occurs, adrenaline 5-10 μg should be given intravenously. If laryngospasm occurs, Succinylcholine 0.5 mg/kg should be given intravenously. Intubation if necessary.

1. Serious: SpO_2_< 70%, and the duration time ≥ 5min after treatment.

*Emergency treatment*: Immediate emergency endotracheal intubation (tracheotomy if necessary) and controlled breathing to deal with the situation of hypoxia. If there is an obstruction of upper respiratory tract, a nasal or oral airway should be placed. If bronchospasm occurs, adrenaline 5-10 μg should be given intravenously. If laryngospasm occurs, succinylcholine 0.5 mg/kg should be given intravenously.

1. Death

- **Arrhythmia**

1. Mild: Atrial premature beats, or junctional premature beats, or single ventricular premature beats ≥ 2 bpm, or junctional escape beats, or degree I atrioventricular block, or degree II atrioventricular block without hemodynamic changes, or mild ST-T changes, and duration time ≥ 30 min.

*Treatment*: Careful observation without medical treatment.

1. Moderate: Atrial fibrillation, or supraventricular tachycardia, or degree II atrioventricular block with hemodynamic changes, or ST-T changes in ≥ 3 leads, and duration time ≥ 30 min.
2. Severe: Grade III atrioventricular block or multiple/frequent ventricular premature beats (≥ 6 times/min or ≥ 30 times/h), or ventricular tachycardia with duration ≥ 20 min.
3. Serious: Ventricular fibrillation or cardiac arrest ≥ 3 min.
4. Death.

*Treatment*: For arrhythmia treatment, cedilanid 0.2 mg iv and observed for 10 min; if it is not relieved after 10 min, repeat it and observe the patient closely for another 10 min. For supraventricular tachycardia, esmolol 40 mg iv and observe the patient for 10 min. If it is not relieved after 10 min, repeat the treatment and closely observe the patient for another 10 min. For degree II atrioventricular block with hemodynamic changes, atropine 0.5 mg iv and observe the patient for 10 min. If it is not relieved after 10 min, repeat it and observe the patient closely for another 10 min. For the degree III atrioventricular block, an isoprenaline infusion at rate 0.03-0.2 μg/kg/min should be administered. Lidocaine 0.5 mg/kg iv should be given for multiple sources of frequent ventricular premature beats or tachycardia, if necessary, repeated administration or dose increase to 1 mg/kg or amiodarone iv. If the symptoms were still not relieved, further treatment measures should be determined according to the clinical situation and consultation with a specialist.

- **Apnea**

1. Mild: SpO2 90-94% after temporary respiratory arrest and assisted ventilation, and duration time ≥ 5 min.

*Treatment*: Careful observation without medical treatment.

1. Moderate: SpO_2_ 80-89% after temporary respiratory arrest and assisted ventilation and the duration time ≥ 5 min after treatment.

*Treatment*: Increase oxygen flow rate to deal with the situation of hypoxia. If there is an obstruction in the upper respiratory tract, a nasal or oral airway should be placed. If bronchospasm occurs, adrenaline 5-10 μg should be given intravenously. If laryngospasm occurs, propofol 2 mg/kg should be given intravenously.

1. Severe: SpO_2_ 70-79% after temporary respiratory arrest and assisted ventilation and the duration time ≥ 5 min after treatment.

*Treatment*: Positive pressure ventilation with mask immediately to deal with hypoxia. If there is an obstruction in the upper respiratory tract, a nasal or oral airway should be placed. If bronchospasm occurs, adrenaline 5-10 μg should be given intravenously. If laryngospasm occurs, succinylcholine 0.5 mg should be given intravenously. Intubation carried out if necessary.

1. Serious: SpO_2_ < 70% after temporary respiratory arrest and assisted ventilation and a duration time ≥ 5 min after treatment.

*Emergency treatment*: Immediate emergency endotracheal intubation (tracheotomy if necessary) and controlled breathing to deal with hypoxia. If there is an obstruction in the upper respiratory tract, a nasal or oral airway should be placed. If the bronchospasm occurs, adrenaline 5-10 μg should be given intravenously. If laryngospasm occurs, succinylcholine 0.5 mg should be given intravenously.

1. Death

- **Involuntary movements**

1. Mild: A small amount of involuntary movement, but did not affect the anesthesia induction procedure.

*Treatment*: Careful observation without medical treatment.

1. Moderate: The subjects showed significant involuntary movement, which affected the anesthesia induction plan.

*Treatment*: For example, limb brake.

1. Severe: The subjects had complications caused by involuntary movement (such as falling out of bed, bruising).

*Treatment*: Deal with complications.

1. Serious: Involuntary movements cause life-threatening risks or require additional hospitalization.

*Emergency treatment*: First aid treatment should be carried out by the researcher according to the clinical situation and relevant departments consulted if required.

1. Death

*Remarks*: Serious AEs and death all belong to serious AEs. If any serious AEs happened, the study would be immediately stopped.

**2. The definition of serious adverse event**

Serious AE refers to a serious medical event occurs during the study and results in the following results:

- **Death**
- **Life threats** (e.g. if necessary interventions are not taken at the time of the event, the patient is at risk of immediate death)
- **Need to be hospitalized or extend the existing length of stay**
- **Permanent or significant loss of function or disability**
- **Cause congenital malformations or defects**
- **Important medical events** (It refers to the situation that although the above results will not occur immediately, it may harm the patient or may require medical or surgical treatment to prevent the patient from having the above results. For example, cancer, bronchospasm requiring intensive treatment in emergency room or at home, malignant blood disease, convulsion not leading to hospitalization, drug dependence, drug abuse, etc)

**Supplementary Table 1.** **Demographic characteristics of the patients (dose escalation and expansion periods)**

| **Induction drug (mg/kg)** | **Ciprofol (mg/kg)** | | | **Propofol (mg/kg)** | | **Total**  **(n = 109)** | ***P*-value** |
| --- | --- | --- | --- | --- | --- | --- | --- |
|  | **0.3 (n = 31)** | **0.4 (n = 8)** | **0.5 (n = 29)** | **2.0 (n = 31)** | **2.5 (n = 10)** |  |  |
| Male / Female, n (%) | 17 / 14 | 4 / 4 | 13 / 16 | 16 / 15 | 5 / 5 | 55 / 54 | 0.966 |
| Age (year), mean ± SD | 44.8 ± 12.7 | 53.5 ± 8.3 | 46.6 ± 12.4 | 44.8 ± 12.4 | 40.2 ± 13.0 | 46.1 ± 12.4 | 0.261 |
| Height (cm), mean ± SD | 163.4 ± 7.2 | 163.6 ± 9.2 | 163.2 ± 8.5 | 164.9 ± 8.4 | 162.8 ± 10.7 | 163.7 ± 8.3 | 0.927 |
| Weight (kg), mean ± SD | 65.1 ± 9.6 | 65.4 ± 9.6 | 63.7 ± 10.7 | 66.4 ± 11.0 | 66.5 ± 14.8 | 65.3 ± 10.7 | 0.888 |
| BMI (kg/m^2^), mean ± SD | 24.4 ± 3 .0 | 24.4 ± 2.5 | 23.8 ± 2.6 | 24.4 ± 3.0 | 24.8 ± 2.7 | 24.2 ± 2.8 | 0.870 |
| ASA status I/II, n | 17 / 14 | 6 / 2 | 7 / 22 | 14 / 17 | 6 / 4 | 50 / 59 | 0.036 |
| Surgical time (min), mean ± SD | 82.2 ± 49.2 | 102.6 ± 44.4 | 81.8 ± 47.9 | 79.8 ± 57.1 | 84.4 ± 35.5 | 83.1 ± 4 9.4 | 0.558 |
| Surgery types |  |  |  |  |  |  |  |
| Abdominal surgery, n | 7 | 1 | 3 | 8 | 7 | 26 |  |
| Urinary surgery, n | 10 | 4 | 10 | 10 | 10 | 44 |  |
| Thyroidectomy, n | 5 | 3 | 1 | 6 | 5 | 20 |  |
| Otorhinolaryngology, n | 6 | 0 | 11 | 4 | 2 | 23 |  |
| Plastic surgery, n | 2 | 0 | 2 | 1 | 0 | 5 |  |
| Gynecologic surgery, n | 1 | 0 | 2 | 1 | 0 | 4 |  |

Note: ASA, American Society of Anesthesiologists physical status; BMI, body mass index; SD, Standard deviation.

**Supplementary Table 2. All the AEs occurred during the entire study period**

- **Adverse events after drug administration in part I study**

| **Adverse Events** | **Ciprofol 0.3 mg/kg**  **n = 9 / 9** | **Ciprofol 0.4 mg/kg**  **n = 8 / 8** | **Ciprofol 0.5 mg/kg**  **n = 8 / 8** | **Propofol 2.0 mg/kg**  **n = 9 / 10** | **Propofol 2.5 mg/kg**  **n = 10 / 10** |
| --- | --- | --- | --- | --- | --- |
| **Cardiac disorders** | | | | | |
| Sinus bradycardia | 3 (33.3%) | 4 (50%) | 3 (37.5%) | 5 (55.6%) | 2 (20%) |
| Sinus tachycardia | 3 (33.3%) | 1 (12.5) | 1 (12.5) | 0 | 3 (30%) |
| I degree atrioventricular block | 0 | 1 (12.5%) | 0 | 0 | 0 |
| **Vascular and lymphatic disorders** | | | | | |
| Hypotension | 0 | 2 (25%) | 2 (25%) | 5 (55.6%) | 3 (30%) |
| Hypertension | 7 (77.8%) | 4 (50%) | 4 (50%) | 4 (44.4%) | 8 (80%) |
| **Musculoskeletal and connective tissue disorders** | | | | | |
| Limbs pain | 0 | 0 | 0 | 0 | 1 (10%) |
| **Skin and subcutaneous tissue disorders** | | | | | |
| Urticaria | 0 | 1 (12.5%) | 0 | 0 | 0 |
| **Nervous system disorders** | | | | | |
| Dizziness | 1 (11.1%) | 0 | 1 (12.5%) | 0 | 3 (30%) |
| Headache | 0 | 0 | 0 | 1 (11.1%) | 1 (10%) |
| Hypoesthesia | 0 | 0 | 0 | 1 (11.1%) | 0 |
| **Gastrointestinal system disorders** | | | | | |
| Vomiting | 1 (11.1%) | 0 | 2 (25%) | 1 (11.1%) | 1 (10%) |
| Nausea | 1 (11.1%) | 0 | 2 (25%) | 1 (11.1%) | 3 (30%) |
| Upper abdominal pain | 0 | 0 | 0 | 0 | 1 (10%) |
| Abdominal pain | 0 | 0 | 0 | 0 | 1 (10%) |
| Dysphagia | 0 | 0 | 1 (12.5%) | 0 | 0 |
| **Systemic disorders and various reactions to the site of drug delivery** | | | | | |
| Fever | 1 (11.1%) | 0 | 0 | 0 | 0 |
| Chill | 1 (11.1%) | 0 | 0 | 1 (11.1%) | 1 (10%) |
| Injection pain | 0 | 0 | 0 | 2 (22.2%) | 1 (10%) |
| **Respiratory system, chest and mediastinal disease** | | | | | |
| Cough | 0 | 0 | 0 | 0 | 1 (10%) |
| Nasal congestion | 0 | 0 | 1 (12.5%) | 0 | 0 |
| Throat irritation | 0 | 0 | 0 | 0 | 1 (10%) |
| Dyspnea | 0 | 0 | 0 | 1 (11.1%) | 0 |
| Dysphonia | 0 | 0 | 1 (12.5%) | 1 (11.1%) | 0 |
| Larynx pain | 0 | 0 | 1 (12.5%) | 0 | 0 |
| **Mental disorders** | | | | | |
| Insomnia | 0 | 0 | 0 | 0 | 2 (20%) |
| **Ear and labyrinthine disorders** | | | | | |
| Earache | 0 | 0 | 0 | 0 | 1 (10%) |
| Vertigo | 0 | 0 | 0 | 0 | 1 (10%) |
| **Various injuries, poisoning and surgical complications** | | | | | |
| Tracheal catheter-induced cough | 0 | 0 | 1 (12.5%) | 1 (11.1%) | 2 (20%) |
| Nausea caused by operation | 0 | 0 | 0 | 1 (11.1%) | 0 |
| Vomiting caused by operation | 0 | 0 | 0 | 1 (11.1%) | 0 |
| **Congenital familial hereditary diseases** | | | | | |
| Fetal chromosomal abnormalities | 0 | 0 | 0 | 0 | 1 (10%) |
| **Medical examination** | | | | | |
| QT interval prolongation  of electrocardiogram | 2 (22.2%) | 2 (25%) | 3 (37.5%) | 2 (22.2%) | 3 (30%) |
| Oxygen desaturation | 2 (22.2%) | 1 (12.5%) | 0 | 1 (11.1%) | 1 (10%) |
| Decreased blood sodium | 0 | 0 | 1 (12.5%) | 0 | 0 |
| Decreased blood calcium | 2 (22.2%) | 0 | 0 | 2 (22.2%) | 0 |
| Decreased blood magnesium | 0 | 0 | 0 | 1 (11.1%) | 0 |
| Decreased blood potassium | 1 (11.1%) | 0 | 1 (12.5%) | 0 | 0 |
| Increased white blood cell count | 2 (22.2%) | 0 | 4 (50%) | 2 (22.2%) | 4 (40%) |
| Decreased percentage of lymphocyte | 2 (22.2%) | 0 | 1 (12.5%) | 0 | 1 (10%) |
| Increased neutrophil percentage | 2 (22.2%) | 0 | 4 (50%) | 0 | 4 (40%) |
| Increased alanine aminotransferase | 1 (11.1%) | 1 (12.5%) | 1 (12.5%) | 0 | 0 |
| Increased aspartate aminotransferase | 1 (11.1%) | 1 (12.5%) | 1 (12.5%) | 1 (11.1%) | 0 |
| Increased ƴ-glutamyltransferase | 0 | 1 (12.5%) | 0 | 0 | 0 |
| Increased blood fibrinogen | 0 | 1 (12.5%) | 0 | 0 | 0 |
| Increased conjugated bilirubin | 0 | 1 (12.5%) | 0 | 0 | 0 |
| Decreased blood albumin | 0 | 0 | 1 (12.5%) | 0 | 0 |
| Decreased total protein | 0 | 0 | 1 (12.5%) | 0 | 0 |
| Increased triglyceride | 0 | 0 | 1 (12.5%) | 0 | 0 |
| Increased lactate dehydrogenase | 0 | 0 | 1 (12.5%) | 0 | 0 |
| Increased α-hydroxybutyrate  dehydrogenase | 0 | 0 | 1 (12.5%) | 0 | 0 |
| Increased total bile acid | 0 | 0 | 1 (12.5%) | 0 | 0 |
| Increased creatine phosphokinase | 0 | 0 | 0 | 1 (11.1%) | 0 |
| Increased blood glucose | 0 | 0 | 0 | 0 | 1 (10%) |
| Increased leukocyte in urine | 2 (22.2%) | 1 (12.5%) | 4 (50%) | 2 (22.2%) | 4 (40%) |
| Positive urine acetone bodies | 3 (33.3%) | 0 | 2 (25%) | 1 (11.1%) | 2 (20%) |
| Positive urine nitrite | 1 (11.1%) | 0 | 0 | 0 | 0 |
| Positive urine protein | 1 (11.1%) | 1 (12.5%) | 3 (37.5%) | 0 | 1 (10%) |
| Positive urine glucose | 2 (22.2%) | 2 (25%) | 0 | 0 | 1 (10%) |
| Increased urinary bacteria | 0 | 1 (12.5%) | 0 | 0 | 2 (20%) |
| Positive urine red blood cell | 0 | 1 (12.5%) | 3 (37.5%) | 0 | 3 (30%) |
| Detection of urine sediment | 0 | 0 | 0 | 0 | 1 (10%) |

- **Adverse events after drug administration in part II study**

| **Adverse Events** | **Ciprofol 0.3 mg/kg**  **n = 22 / 22** | **Ciprofol 0.5 mg/kg**  **n = 21 / 21** | **Propofol 2.0 mg/kg**  **n = 21 / 21** |
| --- | --- | --- | --- |
| **Cardiac disorders** | | | |
| Supraventricular arrhythmia | 0 | 0 | 1 (4.8%) |
| Ventricular extrasystole | 1 (4.5%) | 1 (4.8%) | 0 |
| Bradycardia | 8 (36.4%) | 10 (47.6%) | 12 (57.1%) |
| Palpitations | 0 | 1 (4.8%) | 0 |
| Sinus tachycardia | 3 (13.6%) | 2 (9.5%) | 4 (19.0%) |
| **Vascular and lymphatic disorders** | | | |
| Hypotension | 8 (36.4%) | 10 (47.6%) | 10 (47.6%) |
| hypertension | 14 (63.6%) | 9 (42.9%) | 10 (47.6%) |
| **Skin and subcutaneous tissue disorders** | | | |
| Skin pain | 0 | 1 (4.8%) | 0 |
| **Nervous system disorders** | | | |
| Hypoesthesia | 0 | 0 | 1 (4.8%) |
| Headache | 2 (9.1%) | 0 | 0 |
| Dizza | 3 (13.6%) | 1 (4.8%) | 1 (4.8%) |
| Dyskinesia | 2 (9.1%) | 0 | 0 |
| **Gastrointestinal system disorders** | | | |
| Nausea | 1 (4.5%) | 1 (4.8%) | 3 (14.3%) |
| Upper abdominal pain | 0 | 0 | 1 (4.8%) |
| Vomiting | 3 (13.6%) | 1 (4.8%) | 2 (9.5%) |
| Abdominal pain | 1 (4.5%) | 0 | 0 |
| Loss of appetite | 0 | 1 (4.8%) | 0 |
| **Systemic disorders and various reactions to the site of drug delivery** | | | |
| Fever | 1 (4.5%) | 0 | 0 |
| Chill | 1 (4.5%) | 0 | 0 |
| Chest discomfort | 0 | 1 (4.8%) | 0 |
| Chest pain | 0 | 0 | 1 (4.8%) |
| Injection pain | 2 (9.1%) | 4 (19%) | 5 (23.8%) |
| **Respiratory system, chest and mediastinal disease** | | | |
| Nasal leak | 1 (4.5%) | 0 | 0 |
| Apnea | 0 | 0 | 1 (4.8%) |
| Oropharyngeal pain | 1 (4.5%) | 2 (9.5%) | 2 (9.5%) |
| Sneeze | 1 (4.5%) | 0 | 0 |
| **Various injuries, poisoning and surgical complications** | | | |
| Anesthetic airway complications | 13 (59.1%) | 11 (52.4%) | 10 (47.6%) |
| Pain at the incision | 1 (4.5%) | 2 (9.5%) | 0 |
| Wound complications | 0 | 0 | 1 (4.8%) |
| Wound bleeding | 0 | 0 | 1 (4.8%) |
| **Infections and infectious diseases** | | | |
| Wound infection | 0 | 1 (4.8%) | 0 |
| Postoperative infection | 0 | 1 (4.8%) | 0 |
| Pharyngitis | 1 (4.5%) | 0 | 0 |
| **Hematological and Lymphatic Diseases** | | | |
| Anemia | 0 | 1 (4.8%) | 0 |
| Eosinophilia | 1 (4.5%) | 1 (4.8%) | 0 |
| **Reproductive system and breast diseases** | | | |
| Abnormal penile erection | 1 (4.5%) | 0 | 0 |
| **Medical examination** | | | |
| Oxygen desaturation | 3 (13.6%) | 3 (14.3%) | 2 (9.5%) |
| QT interval prolongation of electrocardiogram | 6 (27.3%) | 10 (47.6%) | 5 (23.8%) |
| ECG ST segment depression | 1 (4.5%) | 0 | 0 |
| Decreased blood potassium | 2 (9.1%) | 3 (14.3%) | 2 (9.5%) |
| Decreased blood magnesium | 1 (4.5%) | 0 | 1 (4.8%) |
| Hypochloremia | 0 | 0 | 1 (4.8%) |
| Decreased blood calcium | 0 | 2 (9.5%) | 1 (4.8%) |
| Decreased blood phosphorus | 1 (4.5%) | 1 (4.8%) | 1 (4.8%) |
| Decreased blood urea | 0 | 1 (4.8%) | 0 |
| Increased white blood cell count | 6 (27.3%) | 4 (19.0%) | 4 (19.0%) |
| Increased mononuclear cell count | 1 (4.5%) | 0 | 1 (4.8%) |
| Decreased anion gap | 1 (4.5%) | 2 (9.5%) | 0 |
| Abnormal hematocrit | 0 | 1 (4.8%) | 0 |
| Decreased percentage of lymphocyte | 2 (9.1%) | 3 (14.3%) | 0 |
| Decreased lymphocyte count | 1 (4.5%) | 0 | 0 |
| Increased neutrophil percentage | 1 (4.5%) | 1 (4.8%) | 0 |
| Decreased platelet count | 2 (9.1%) | 0 | 0 |
| Increased blood glucose | 2 (9.1%) | 2(9.5%) | 1(4.8%) |
| Increased C-reactive protein | 5(22.7%) | 4(19.0%) | 6(28.6%) |
| Increased alanine aminotransferase | 0 | 2(9.5%) | 1(4.8%) |
| Increased aspartate aminotransferase | 0 | 2(9.5%) | 1(4.8%) |
| Decreased HDL | 0 | 1(4.8%) | 0 |
| Decreased RBC | 0 | 1(4.8%) | 1(4.8%) |
| Increased conjugated bilirubin | 0 | 0 | 2 (9.5%) |
| Increased unbound bilirubin | 0 | 0 | 1 (4.8%) |
| Increased γ-glutamyltransferase | 0 | 1 (4.8%) | 0 |
| Increased blood alkaline phosphatase | 0 | 1 (4.8%) | 0 |
| Increased leukocyte in urine | 7 (31.8%) | 4 (19.0%) | 6 (28.6%) |
| Positive urine protein | 3 (13.6%) | 3 (14.3%) | 5 (23.8%) |
| Positive urine red blood cell | 3 (13.6%) | 5 (23.8%) | 5 (23.8%) |
| Positive urine nitrite | 0 | 0 | 1 (4.8%) |
| Positive urine glucose | 1 (4.5%) | 0 | 0 |
| Positive urine acetone bodies | 6 (27.3%) | 2 (9.5%) | 8 (38.1%) |
| Increase prothrombin time | 0 | 1 (4.8%) | 0 |
| Decreased blood albumin | 3 (13.6%) | 2 (9.5%) | 3 (14.3%) |
| Decreased haemoglobin | 0 | 0 | 1 (4.8%) |
| Decreased total protein | 2 (9.1%) | 2 (9.5%) | 2 (9.5%) |
| Increased triglyceride | 1 (4.5%) | 1 (4.8%) | 1 (4.8%) |
| Increased blood creatine phosphokinase | 5 (22.7%) | 3 (14.3%) | 4 (19%) |
| Increased serum creatinine | 0 | 1 (4.8%) | 0 |
| Increased urobilinogen | 0 | 0 | 1 (4.8%) |
| Decreased prealbumin | 0 | 1 (4.8%) | 0 |
| Detection of urine sediment | 2 (9.1%) | 1 (4.8%) | 1 (4.8%) |
